# Drinking by sea snakes from oceanic freshwater lenses at first rainfall ending seasonal drought

**DOI:** 10.1371/journal.pone.0212099

**Published:** 2019-02-07

**Authors:** Harvey B. Lillywhite, Coleman M. Sheehy, Mark R. Sandfoss, Jenna Crowe-Riddell, Alana Grech

**Affiliations:** 1 Department of Biology, University of Florida, Gainesville, Florida, United States of America; 2 Florida Museum of Natural History, University of Florida, Gainesville, Florida, United States of America; 3 ARC Centre of Excellence for Coral Reef Studies, James Cook University, Townsville, Queensland, Australia; Department of Agriculture and Water Resources, AUSTRALIA

## Abstract

Acquisition of fresh water (FW) is problematic for FW-dependent animals living in marine environments that are distant from sources of FW associated with land. Knowledge of how marine vertebrates respond to oceanic rainfall, and indeed the drinking responses of vertebrates generally following drought, is extremely scant. The Yellow-bellied Sea Snake (*Hydrophis platurus*) is the only pelagic species of squamate reptile and ranges across the Indo-Pacific oceans, having one of the largest geographic distributions of any vertebrate species. It requires FW and dehydrates at sea during periods of drought. Here we report drinking behaviors of sea snakes precisely at the transition from dry to wet season when rainfall first impacted the ocean following 6 months of seasonal drought. We show that the percentage of sea snakes that voluntarily drank FW in the laboratory when captured over eight successive days decreased from 80% to 13% before and after rainfall commenced, respectively. The percentage of snakes that drank immediately following capture exhibited a significant linear decline as the earliest rains of the wet season continued. Drinking by snakes indicates thirst related to dehydration, and thus thirsty snakes must have dehydrated during the previous six months of drought. Hence, the progressive decline in percentage of thirsty snakes indicates they were drinking from FW lenses associated with the first rainfall events of the wet season. These data reinforce the importance of accessing oceanic FW from precipitation, with implications for survival and distribution of pelagic populations that might be subjected to intensifying drought related to climate change.

## Introduction

Marine environments present extremely harsh challenges for secondarily marine vertebrates owing to the general absence of fresh water (FW). Hence, evolutionary transitions from terrestrial or freshwater habitats to marine environments are difficult [1–3]. Many secondarily marine vertebrates are thought to live independently from direct sources of FW [4]. Sea snakes are especially interesting because they have salt glands and were formerly thought to drink seawater. However, recent studies indicate that the principal clades of marine snakes—laticaudine sea kraits and hydrophiine sea snakes (the most diverse and speciose marine reptiles)—remain dependent on FW, and this is manifest in their abundance and distribution [5–11].

Acquisition of FW is problematic for sea snakes living in oceanic environments that are distant from sources of FW associated with land. These include species living on reefs that are far from land, and especially waifs or pelagic snakes in the open ocean. A uniquely interesting species of sea snake is *Hydrophis platurus* (Yellow-bellied Sea Snake), which is the only pelagic species of squamate reptile and ranges from southern Africa across the Indo-Pacific to the Pacific coast of Central America [12–14]. It is subject to long-range movements by drifting with gyres and currents [15], and it is the only species of sea snake to have reached the Americas. Recent investigations show that, like other sea snakes investigated, this species is dependent on FW [9] and dehydrates at sea during periods of drought [10]. Snakes captured during the end of the dry season exhibit lower body condition and lower content of total body water than do snakes captured during the end of the wet season [10]. This would not be the case if these snakes could remain hydrated by drinking seawater as formerly supposed.

The dehydration and drinking behavior of *H*. *platurus* has been followed in the Golfo de Papagayo of northwestern Guanacaste, Costa Rica, since 2009 [9, 10]. This region experiences seasonal drought generally lasting from December to May or June, and hence snakes do not have access to FW from rainfall for possibly 6–7 months of the year. Because of its pelagic marine habitat, the only source of FW for this snake is rainfall that forms a FW or dilute brackish-water lens during intense precipitation over the ocean. Knowledge of how marine vertebrates respond to oceanic rainfall, and indeed the drinking responses of vertebrates generally following drought, is extremely scant [16].

Here we show that pelagic sea snakes drink FW from FW lenses that form during heavy rainfall at the start of the wet season following 6 months of seasonal drought. These findings confirm that sea snakes dehydrate during drought and rehydrate using FW lenses formed from rainfall as soon as the wet season begins. Such attributes have implications for the survival and persistence of this unique species of sea snake that is distributed over 2/3 of the Earth’s circumference and is likely sensitive to changes in precipitation that may be related to climatic change.

## Materials and methods

### Ethics statement

This research was conducted within guidelines and approval of the University of Florida IACUC, approval 201502798. Permission to collect sea snakes was obtained under permit resolución No. ACT-OR-DR-055-17 from the Area de Conservación Arenal Tempisque (ACT) del Sistema Nacional de Areas de Conservacion (SINAC), Costa Rica.

### Drinking observations

**Fig 1:**
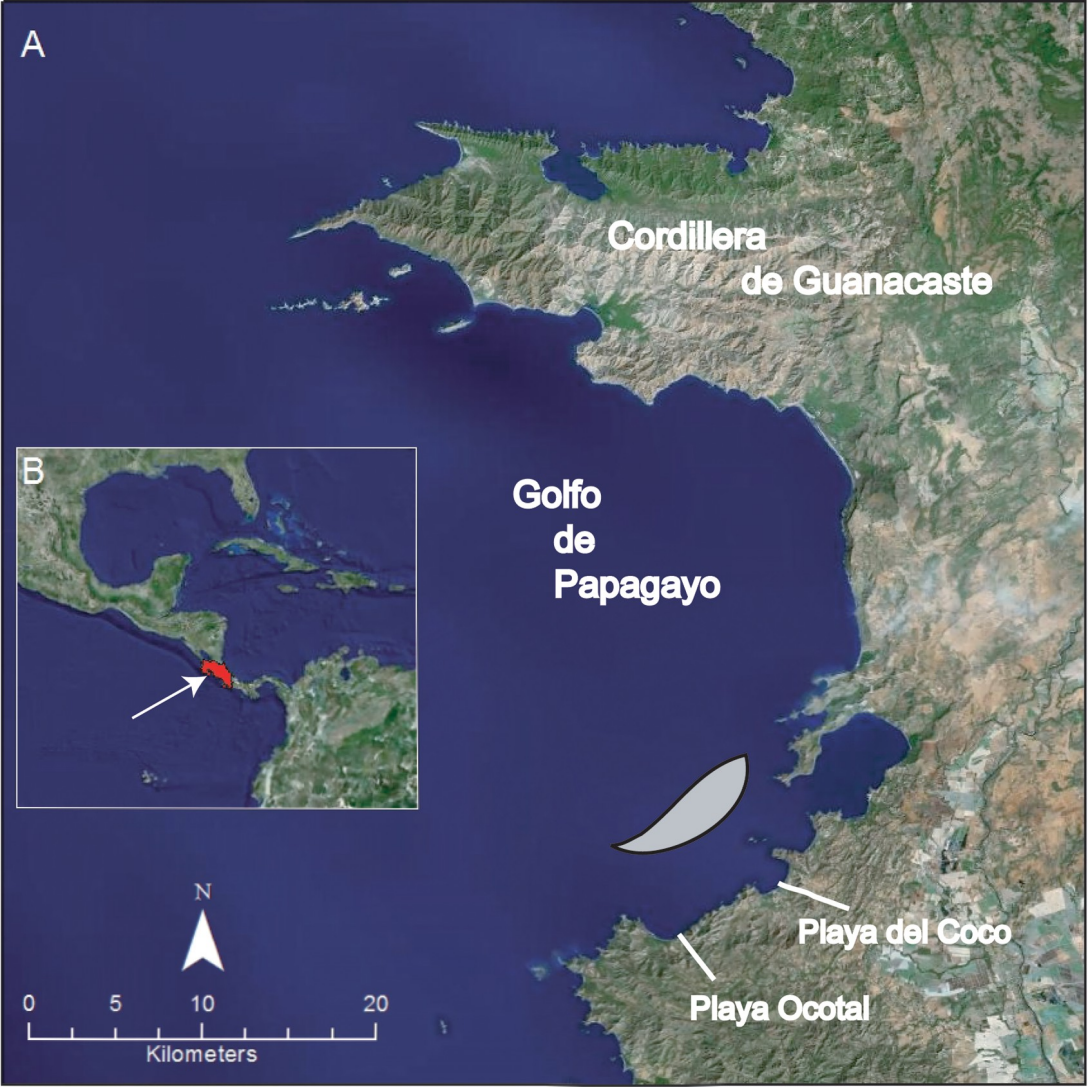
Map of study area in Costa Rica. A. Northwest coast of Guanacaste showing location of Golfo de Papagayo and beaches near the offshore area where sea snakes were collected (shown in gray). B. Inset showing location of Costa Rica in Central America with arrow pointing to the area of study offshore at Guanacaste.

During a research trip to Costa Rica from 5 to 14 May 2017, we captured sea snakes and immediately tested them for thirst and drinking precisely during the climatic transition from dry to wet season in the coastal waters of Guanacaste (Fig 1). When we arrived in Costa Rica, there had not been significant rainfall at the location where snakes were studied; the weather was hot and dry, and there was no precipitation over the ocean. Although *H*. *platurus* is a pelagic species of sea snake, very little is known about the movement of individuals in and out of populations. At least one population in Golfo Dulce, Costa Rica, appears to be relatively stable and static in location [13]. Here we have assumed that, depending on the movement of currents, it is likely that individual snakes spend considerable time at localities associated with seasonal drought at Guanacaste, Costa Rica.

We captured a total of 99 *H*. *platurus* during eight consecutive mornings of daily sampling, except for May 11 when insufficient snakes were located to be included in the drinking experiments. On all other days, snakes were collected 3–6 km offshore (latitude 10.556 to 10.620, longitude 85.691 to 85.752; Fig 1) during morning hours beginning approximately 0700 h local time. Each snake was captured individually using a handheld dip net while it floated in a “float and wait” posture on the ocean surface [17]. Snakes were temporarily held in mesh nets within an insulated cooler and returned immediately to the laboratory where they were weighed after brief exposure to room air while lying on a dry cloth towel until the skin was dry to the touch. After weighing, each snake was placed individually in a plastic container (sweater or shoe box) half-filled with FW. Snakes were held in FW for approximately 20 h, reweighed, and released before another sample was collected from the ocean [9, 10].

We observed drinking behaviors of freshly-captured snakes for up to one hour after they were first placed in FW where they were held overnight. The amount of FW that each snake ingested was quantified by changes in mass that occurred overnight. Each snake was weighed inside a tared container on a Sartorius ELT2001 electronic top-loading balance before being placed in FW, and the snake was weighed again the following morning approximately 20 h later. Before each weighing, the snake was placed on a cloth towel and its skin allowed to dry in air. We have used these procedures to quantify drinking in sea snakes previously [5, 7, 9, 10, 11].

Following these drinking trials, snakes were released in the Golfo de Papagayo, and another sample of animals was captured and returned to the laboratory. Snakes were released distant from the original point of capture. During previous work in Costa Rica, we could identify snakes by tail clippings that were harvested for genetic analyses ([13] and unpublished). During the earlier daily sampling of snakes similarly to what we report here [see 10], we recaptured only a single individual out of hundreds that were released in the Golfo de Papagayo (HBL and CMS III, unpublished observations) where these snakes are extremely abundant [18]. Hence we are confident that daily captures of snakes in the present study represent new individuals different from those we released at other locations.

### Precipitation estimates from satellite and salinity of ocean water

**Fig 2:**
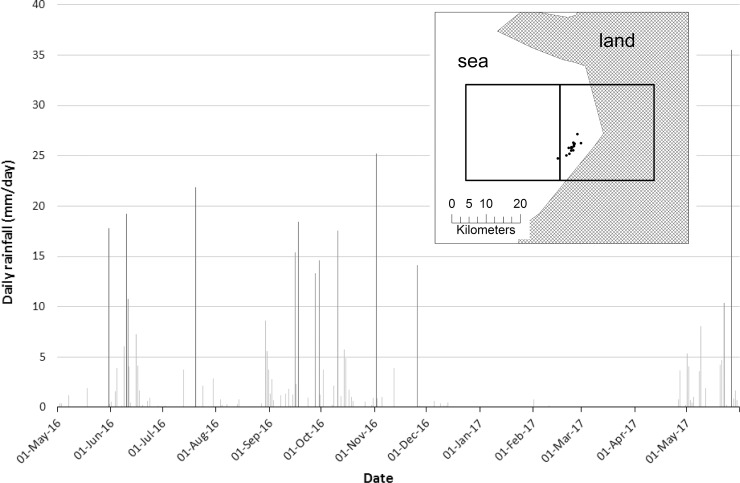
Daily rainfall for the year May 2016 to May 2017. Vertical bars quantify daily rainfall and illustrate the period of drought roughly from December 2016 to May 2017. For presentation, we calculated the mean daily rainfall (mm) from CMORPH data [19] of two grid values represented by boxes and the area shown on the inset map. The black dots represent GPS locations where sea snakes were collected. The area shown in grey represents land, and the ocean is shown in white. Note that land as well as sea is represented in part of the right-hand grid.

To complement our direct observations of rainfall, we determined the pattern of precipitation from satellite data for the area and times that we sampled. Daily rainfall measures of the study region were derived from NOAA CPC Morphing Technique (CMORPH) Global Precipitation Analyses Version 0.x [19]. CMORPH produces global precipitation analyses at a resolution of 0.25 degree (~ 27 kilometres). The CMORPH data were mapped and analyzed in the geographic information system (GIS) software ArcGIS 10.4.1. Daily rainfall (mm) from 1^st^ May 2016 to 31^st^ May 2017 was calculated as the mean of the two 0.25-degree grid cells that covered the area where sea snakes were collected (Fig 2).

Each morning that we collected snakes for drinking investigations we took water samples at the location where snakes were captured. The salinity of water samples was measured using a portable salinity refractometer.

### Statistics

All measurements are expressed as means ± SEM. Variation and trends in data were analyzed using linear regression, ANOVA, and Mann-Whitney U test. Statistical analyses were conducted using Statview SAS 5.0.1.

## Results

**Fig 3:**
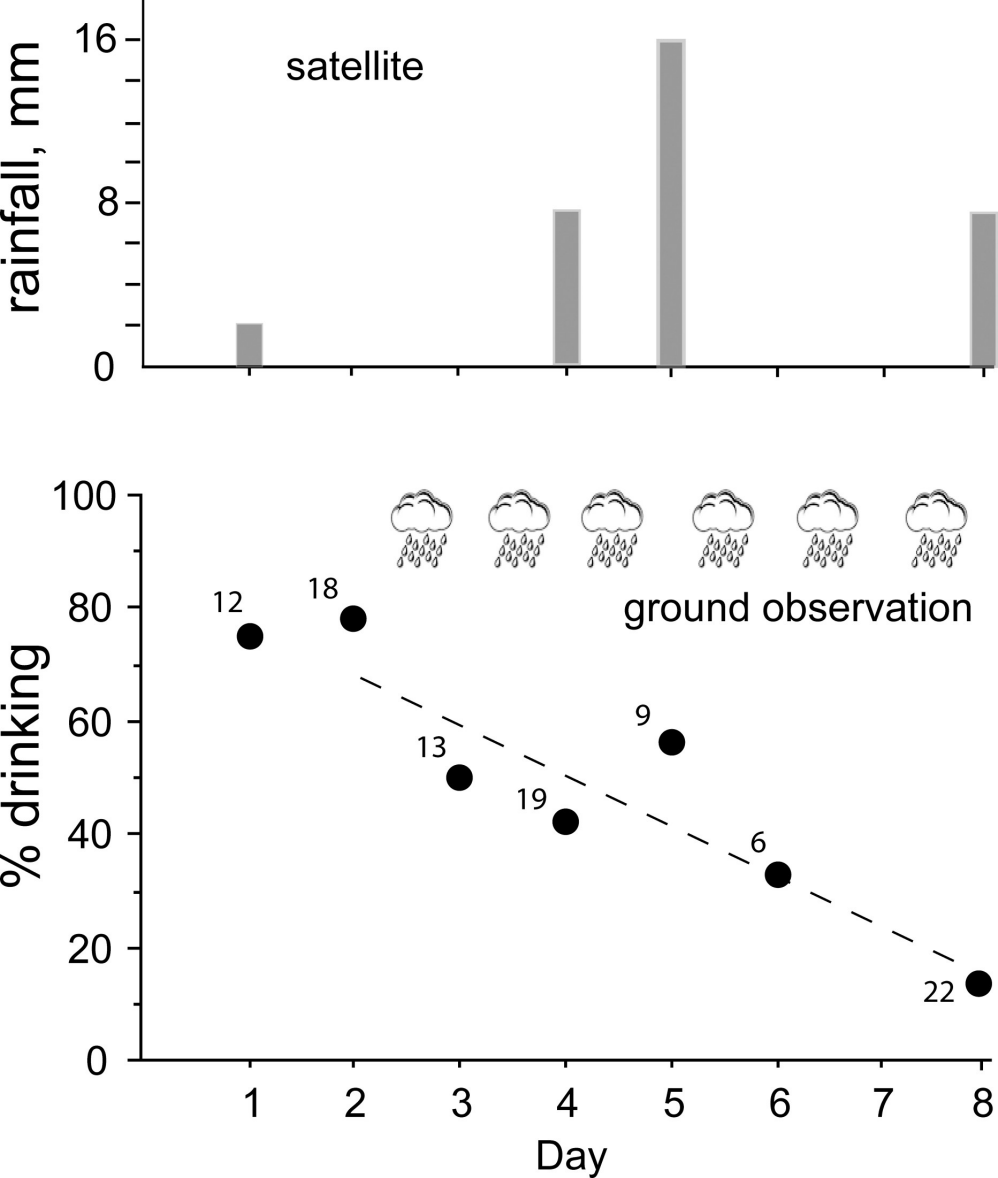
The percentage of sea snakes (*Hydrophis platurus*) that voluntarily drank fresh water immediately following capture on different days beginning at the end of the dry season at Golfo de Papagayo, Costa Rica. These percentages are plotted in relation to the beginning of the very first storms of the wet season that produced heavy rainfall over the ocean where snakes were collected. The cloud symbols represent heavy rainfall that was observed directly over the ocean during the afternoon or evening beginning just before the third day of sampling. The small numbers next to each data point represent the numbers of snakes in each sample. The upper graph illustrates the daily rainfall as determined from NOAA CPC Morphing Technique (CMORPH) Global Precipitation Analyses Version 0.x [19]. The CMORPH data likely do not reflect accurately the precipitation that was observed because of the low spatial resolution relative to the area of water where we collected snakes (see Figs 1 and 2). The right-hand grid cell includes land area as well as ocean, hence the small amount of rainfall on Day 1 likely reflects precipitation we observed over land but not the ocean, and therefore not accessible to *H*. *platurus*. Following the advent of significant precipitation that we observed over the ocean (cloud symbols), the linear decline in number of snakes drinking fresh water in the laboratory was significant (dashed line in lower graph; Y = 85.819–8.7729X, *R*^2^ = 0.851, *P* = 0.0031).

**Fig 4:**
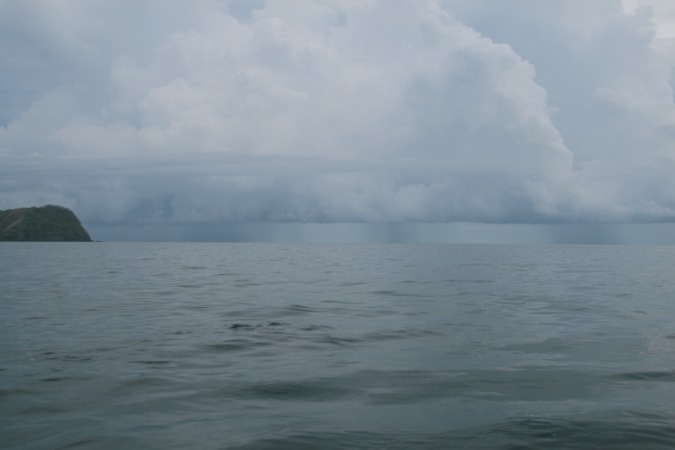
Three discrete points of rainfall during afternoon thunderstorms observed on 12 May 2017, Golfo de Papagayo, Guanacaste, Costa Rica. The photograph illustrates how rainstorms over the ocean can produce patchy opportunities for pelagic snakes to drink fresh water. Photograph by H.B. Lillywhite.

**Fig 5:**
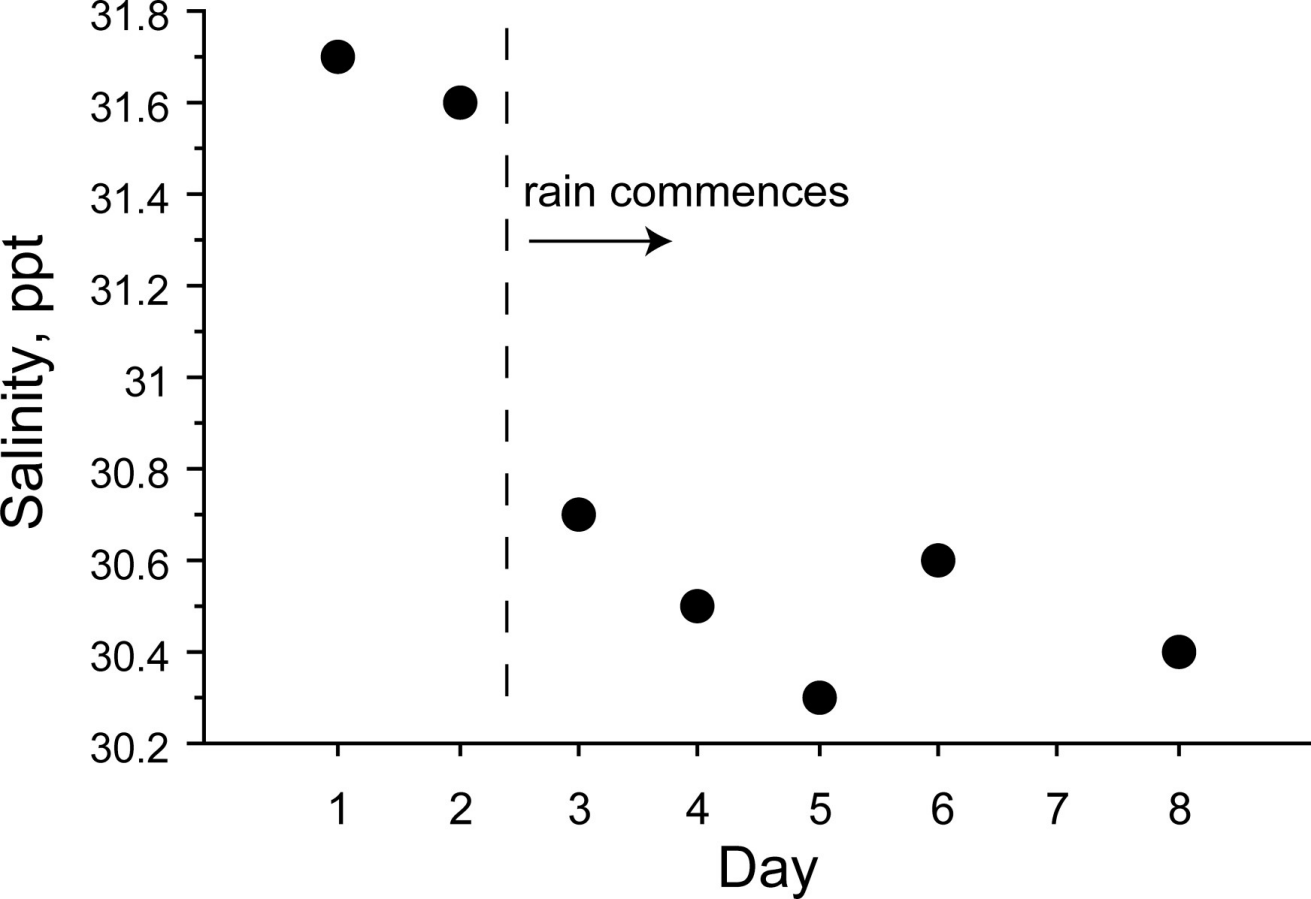
Salinity of ocean surface waters where snakes were collected. Data points represent salinity of water measured on each day that snakes were collected, and the vertical dashed line indicates the onset of wet-season rainfall.

A large proportion of snakes (~ 80%) that were captured during the first two days drank FW soon after they were returned to the laboratory (Fig 3). Many of these snakes were observed drinking during the first hour of observation, while others drank overnight (determined by increases in mass). Between the second and third day of sampling there was heavy rainfall during the afternoon, and we directly observed that rain fell in large amounts over the Golfo de Papagayo near Playa del Coco and Ocotal where snakes were collected (Fig 4). During the next several days, precipitation occurred during the afternoon or evening, and again we observed heavy rain falling directly over the ocean. Once rainfall began at the beginning of the wet season, the salinity of surface water decreased in the Golfo Papagayo during the eight days of our investigations (Fig 5).

The percentage of snakes drinking FW soon after capture decreased with time, falling from ~ 80% before the rains began to 13% on the eighth day (Fig 3). A linear regression model fitted to log-transformed data confirms that the decrease in number of snakes drinking was significant (Y = 2.052–0.098X, *R*^2^ = 0.842, *P* = 0.0036).

**Fig 6:**
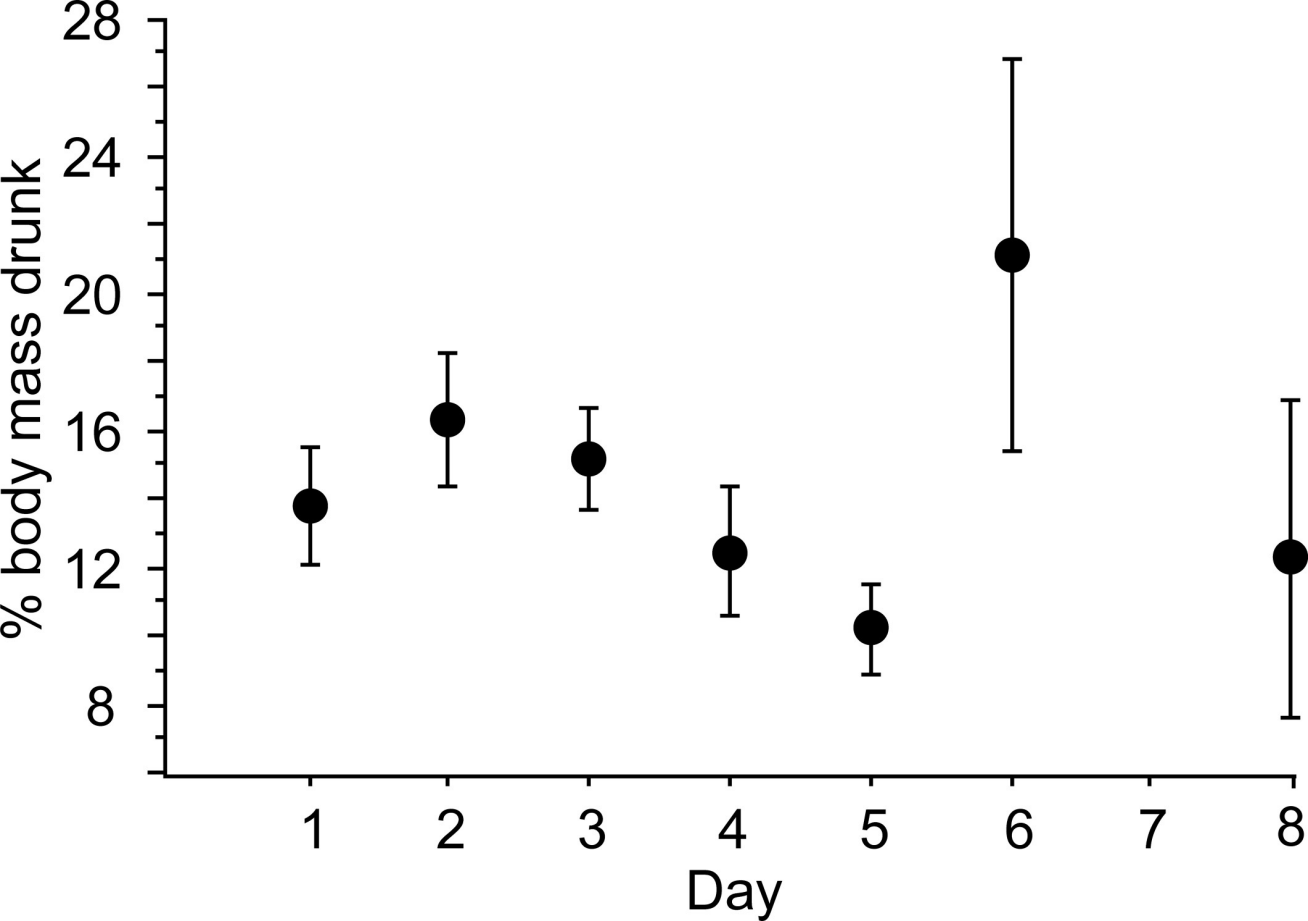
Relative amount of fresh water that was drunk by snakes captured on different days at the transition of dry to wet season (days 2–3) at Golfo de Papagayo, Guanacaste, Costa Rica. Data are displayed as means ± SEM. Sample sizes can be determined from Fig 3. The variation in amount of water drunk was not significant across the span of daily measurements (ANOVA, F_6, 40_, P = 0.255).

There was no clear pattern with respect to the amount of FW that was consumed by drinking snakes. The volume of FW that was drunk by snakes ranged from ~ 8% to ~ 27% of body mass, but did not vary with respect to time since first measurements or first rainfall (ANOVA, F_6, 40_, P = 0.255; Fig 6). There was no significant correlation between body mass and the amount of water that snakes drank (*r* = 0.034, P = 0.823). However, the mean size of snakes drinking (mass = 35.97 ± 3.11 g) was smaller than that of snakes not drinking (mass = 51.15 ± 4.87 g) (Mann-Whitney U test, Z = − 2.092, P = 0.0365).

## Discussion

Our data clearly indicate that within a few days of the advent of the wet season, a decreasing number of sea snakes were in negative water balance as rainfall created opportunities for drinking FW at the ocean’s surface in the study area. Thus, it is appropriate to conclude that thirsty snakes, having accumulated a water deficit during the previous dry season, commenced drinking from FW (or dilute brackish-water) oceanic lenses when precipitation began to fall over the Golfo de Papagayo. Short of directly observing a snake drinking from an oceanic lens during intense storms and rainfall (difficult at best), data reported here are the strongest evidence to date that sea snakes utilize FW lenses as critical resources of FW [20, 21]. These data are the result of the serendipitous opportunity to quantify drinking behaviors of sea snakes precisely at the transition from dry to wet season when rainfall first impacted the ocean at our location. Other possible sources of fresh water following rainfall might be estuaries at various locations along the coastline. However, in many years of collecting and sighting sea snakes along the Guanacaste coast, we have always observed snakes out in the open ocean and have never seen a single snake in the estuaries we have examined for their presence.

Our observations of thirst in a pelagic sea snake at the onset of wet season is parallel to observations of Bonnet and Brischoux [16] who reported that numerous sea kraits emerged from terrestrial refugia and drank from small rock pools following rainfall that ended a drought. Hence, thirsty snakes—terrestrial or marine—‘binge’ drink from temporary sources of fresh water made available from precipitation following drought. Our data for mean size of snakes drinking suggest that smaller individuals are more sensitive to dehydration than are larger individuals. However, there was no correlation of body size with the amount of water that was drunk. This latter finding is consistent with the variability of drinking volumes characteristic of this and other species of marine snakes [5, 9].

In laboratory tests, *H*. *platurus* withstand experimental dehydration ranging from 10 to 26% of body mass before being stimulated to drink FW, and the rates of dehydration in seawater are slow [9]. Thus, snakes at sea can be expected to be in variable states of dehydration, and thus show variable tendencies to drink FW [10]. Moreover, once rains begin at the onset of the wet season, sea snakes at different locations might have variable opportunities to drink FW because of the spatial heterogeneity of storms. The intensity of storms also is important, as well as mixing conditions that might produce variable conditions of FW lenses. *Hydrophis platurus* in the laboratory voluntarily drink brackish water up to about 50% seawater [9]; however, snakes drinking brackish water will tend to repeat drinking at smaller volumes if subsequently offered repeated access to FW. Nonetheless, our data demonstrate that a high percentage of snakes are at, or have surpassed, the threshold for thirst and drinking FW at the beginning of the wet season, and this percentage drops rather sharply as seasonal rains continue over the ocean. Thus, thirsty snakes replenish their body water when precipitation provides access to FW following drought.

The volumes of FW ingested by snakes captured from the ocean generally match those of snakes in previous laboratory tests (mean ~ 13% body mass, [9]). The fact that drinking volumes were statistically invariant across days suggests that the snakes we captured were in similar states of dehydration and did not represent individuals that were drinking repetitively over the eight days of study.

These data reinforce the importance of seasonal availability of FW for pelagic sea snakes, which dehydrate in the ocean and are thus susceptible to drought [10]. Previous assumptions about sea snakes drinking seawater (*e*.*g*., [4]) were likely based in the fact that sea snakes possess salt glands. However, Dunson and Dunson [22] described the sublingual salt gland of sea snakes as being small with comparatively low rates of secretion. Thus, it seems that while salt glands of sea snakes might assist water and ion balance, they evidently cannot by themselves enable sea snakes to drink sea water to avoid dehydration [5].

Insofar as sea snakes require FW to remain in water balance, species that are dependent on FW lenses formed from rainfall are potentially susceptible to drought, even while having evolved considerable tolerance for dehydration [9, 10]. Although not yet investigated, *H*. *platurus* could also provide important insight concerning how drought or salinity influences movement ecology of species that are susceptible to, or voluntarily respond to, drifting with changes of currents [8, 13, 15]. Climate change is expected to increase the extent and intensity of drought in the tropics [23, 24], and may also promote movements or changes of geographic ranges of some species [14]. We further suggest it might be useful to investigate the drinking behaviors of other marine organisms such as sea turtles, fishes, and crabs, because the assumptions for drinking seawater without a requirement for FW are unproven for many species (and life stages) that have not been investigated in the laboratory.

## Supporting information

S1 Data Data. (XLSX)
